# Optimizing the blending ratio of urea with slow-release N fertilizer improves maize yield and N use efficiency while reducing N losses

**DOI:** 10.3389/fpls.2026.1895413

**Published:** 2026-07-28

**Authors:** Nv Zhang, Jinjin Guo, Qichang Ma, Yong Yuan, Pengzhou Yin, Zhizhao Lin, Xiangtong Zeng, Xiaogang Liu, Fucang Zhang

**Affiliations:** 1 Yunnan Key Laboratory of Efficient Utilization of Agricultural Water Resources and Intelligent Control, Faculty of Modern Agricultural Engineering, Kunming University of Science and Technology, Kunming, China; 2 Faculty of Modern Agricultural Engineering, Yunnan International Joint Laboratory of Intelligent Agricultural Engineering Technology and Equipment, Kunming University of Science and Technology, Kunming, China; 3 Faculty of Modern Agricultural Engineering, Seasonal Arid Region Water-Soil-Crop System Observation and Research Station of Yunnan Province, Kunming University of Science and Technology, Kunming, China; 4 Key Laboratory of Agricultural Soil and Water Engineering in Arid and Semiarid Areas of the Ministry of Education, Northwest A&F University, Xi’an, China

**Keywords:** fertilizer type, maize cultivar, N loss, N uptake, N use efficiency

## Abstract

**Introduction:**

Blending urea (U) with slow-release N fertilizer (R) can improve the synchrony between N supply and crop demand, while mitigating N losses to the environment. However, cultivar-specific responses to varied U/R blending ratios under rainfed conditions remain poorly characterized.

**Methods:**

In this study, two dominant maize cultivars (ZD958 and QL14) widely planted in Northwest China were selected as experimental materials. Five fertilization treatments (U, R, and three U/R blending ratios: UR1, UR2, UR3) each at two N rates (N1: 180 kg·ha^−1^; N2: 240 kg·ha^−1^) were tested.

**Results:**

The results showed that UR treatments generally achieved higher grain yield and N use efficiency (NUE), while reducing cumulative NH_3_ volatilization and soil NO_3_^-^–N residue compared with U and R. For both ZD958 and QL14, the maximum grain yield and NUE were achieved under the UR2N1 (U/R = 3/7, 180 kg·ha^−1^). Compared with other treatments, UR2N1 increased NUE by 3.51–19.07% (ZD958) and 4.01–20.41% (QL14), while reducing NH_3_ volatilization soil NO_3_^-^–N residue. Notably, under UR2N1, QL14 showed 9.70% higher NUE than ZD958, along with 8.78% lower soil NO_3_^-^–N residue and 14.12% lower NH_3_ volatilization.

**Conclusion:**

Under rainfed conditions, the UR2N1+QL14 combination effectively balances the trade-off between grain yield and environmental impact, and is therefore recommended as an optimal fertilization–cultivar strategy for rainfed maize production in Northwest China.

## 1 Introduction

Nitrogen (N) fertilizer serves as an indispensable N source for crop growth and development, and substantially improves crop yield (Jiang et al., 2024). Driven by the continuous growth of the global population, the worldwide food demand is increasing steadily, leading to the prevalent over-application of N fertilizers in agricultural systems (Congreves et al., 2021). Excessive N input not only restricts crop growth but also triggers a series of ecological and environmental issues, including soil salinization, soil fertility degradation, and greenhouse gas emissions (Wang et al., 2024). Previous studies have demonstrated that agricultural activities contribute approximately 90% of global ammonia (NH_3_) emissions (Wyer et al., 2022). Particularly summer maize cultivation is highly susceptible to intensive NH_3_ volatilization, such as high temperatures and abundant rainfall during the growing season accelerate the decomposition of N fertilizers, further aggravating soil NH_3_ volatilization and regional agricultural NH_3_ emissions (Yang et al., 2020). Accordingly, implementing scientific N fertilizer management strategies can help reduce excessive N application, effectively mitigate N environmental losses, and guarantee the sustainable development of food production (Dimkpa et al., 2020).

Urea (U) accounts for approximately 70% of global N fertilizer use due to its high N content and relatively low cost (Zhang et al., 2022a). Therefore, multiple topdressing of U has been widely recognized as an effective strategy to improve N use efficiency (NUE) by synchronizing soil N supply with crop N requirements across different growth stages. However, this fertilization method inevitably increases time and labor costs in agricultural production (Zhang et al., 2022b). With the development of modern agricultural technologies, slow-release N fertilizer (R) has emerged as a promising alternative to address the aforementioned drawbacks (Priya et al., 2024). R possess a unique slow-release characteristic that can precisely match crop nutrient demand in terms of nutrient quantity, release timing and spatial distribution throughout the growing season, while improving soil N supply capacity (Wang et al., 2021). However, the large-scale popularization and application of R fertilizers are constrained by obvious limitations. On the one hand, the high production cost of R restricts their widespread field adoption (Kyebogola et al., 2024). On the other hand, their nutrient release dynamics are highly susceptible to multiple factors, including coating materials, soil conditions (e.g., soil temperature), and environmental factors (e.g., rainfall) (Fan et al., 2011; Lawrencia et al., 2021). Considering the rapid soil dissolution of U, the insufficient N supply of R at the early crop growth stage, and the high application cost of pure R, the blended application of urea and slow-release N fertilizer (UR) has been proposed as a feasible optimized fertilization strategy (Zheng et al., 2020). Studies have verified that one-time basal application of UR simplified the fertilization process, reduces agricultural costs, and supplements N demand during the later growth stages of crops (Andrade et al., 2021). Previous findings have also demonstrated that UR blending treatments with 45–70% slow-release N proportion can significantly reduce soil residual NO_3_^-^–N, while effectively improving crop grain yield and NUE (Garcia et al., 2018). Therefore, this study aims to explore the optimal UR blending ratio to achieve better synchronization between soil N supply and crop N demand throughout the entire maize growing season.

In addition, genotypic variation among maize cultivars has been widely recognized as a core determinant of differential NUE (Ren et al., 2023). Maize genotypes exhibit substantial discrepancies in root morphological traits, physiological characteristics, and N uptake kinetics, which collectively determine root nutrient acquisition capacity (Ibn et al., 2024). In addition to directly influencing nutrient uptake, cultivar-specific root traits can modulate the structure of root-associated microbial communities, thereby regulating soil N transformation processes and ultimately driving variations in maize NUE (Li et al., 2022). Moreover, genotypic differences in drought tolerance, disease resistance, and other stress-adaptive traits can constrain crop nutrient uptake, disrupt normal plant growth, and consequently alter grain yield performance (Miao et al., 2022). Therefore, screening and adopting high-N-efficient maize cultivars is critical for stabilizing crop productivity and guaranteeing regional food security (Wang et al., 2023).

Rainfed farming constitutes the dominant agricultural production system in Northwest China. Maize serves as the primary staple cereal crop and a major source of household economic income in this region. To pursue higher grain yields, excessive N fertilization has become a common practice in local maize production, resulting in severe soil N losses and substantial ecological environmental risks (Guo et al., 2024). Previous studies have confirmed that blended application of urea and slow-release N fertilizer can effectively reduce N input rates, mitigate environmental N losses, and improve crop productivity (El-Sharkawy et al., 2024). Nevertheless, two critical knowledge gaps remain unclear for rainfed maize cultivation in Northwest China: the optimal U/R blending ratio for local field conditions, and whether the N release patterns of different UR combinations can synchronize with the N uptake characteristics of different maize cultivars to simultaneously achieve high grain yield and high NUE. Therefore, optimizing the UR blending ratio and screening the matching maize cultivar is essential for synergistically improving yield and N use efficiency under regional rainfed conditions. We hypothesized that, under rainfed conditions in Northwest China, the coordination of an optimized U/R blending ratio and genotype-specific N uptake traits can achieve the dual goals of high maize yield and high NUE. Therefore, the main objectives of this study were: (1) to evaluate the effects of different U/R blending ratios under two N application rates on grain yield, NUE, and N losses characteristics of two widely cultivated maize cultivars (ZD958 and QL14); 2) to determine the optimal U/R blending ratio for rainfed maize production in Northwest China; 3) to clarify the superior maize cultivar with higher yield and NUE performance under the optimal fertilization regime.

## 2 Materials and methods

### 2.1 Experimental site

**Figure 1:**
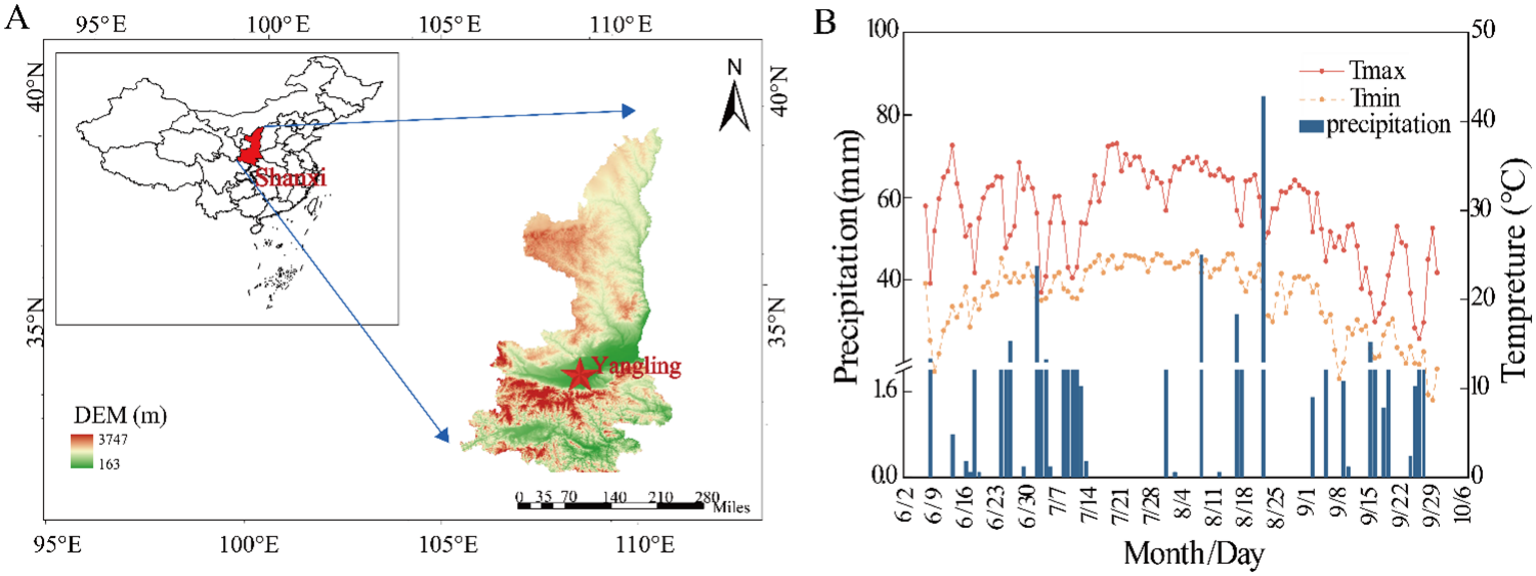
Accurate position of maize experimental field **(A)** and daily temperature and precipitation during the maize growing season **(B)**.

The field experiment was conducted from 7 June to 30 September 2018 at the Water-saving Station of the Key Laboratory of Agricultural Soil and Water Engineering in Arid and Semiarid Areas, Northwest A&F University, Shaanxi Province, China (34°18′N, 108°24′E; 521 m.a.s.l.). The region has a warm temperate monsoon climate and is characterized as semi-humid but drought-prone. The long-term mean annual precipitation is 562.90 mm, with most rainfall occurring from July to September (**Figure 1**). The mean annual temperature is 18.41 °C, and the average relative humidity is approximately 47.44%. The soil texture in the 0–20 cm layer was classified as silty clay loam based on laser particle analysis. The basic physicochemical properties of the topsoil (0–20 cm) are presented in **Table 1**. Meteorological data for 2018 were compared with historical records for the same region (**Supplementary Figure 1**) to provide climatic context for the experimental year.

**Table 1:** The physical and chemical properties of soil in 0–20cm soil layer.

Soil properties	Value	Measurement method
pH	8.14	Acid–alkali indicator method
Soil texture	silty clay loam	Laser particle size analyzer
Total N	0.89 g·kg−1	Kjeldahl digestion
Total phosphorus	0.60 g·kg−1	Molybdenum antimony colorimetric method
Total potassium	14.10 g·kg−1	Emission spectrophotometry method
Available phosphorus	8.28 mg·kg−1	Olsen method by 0.5 mol·L−1 NaHCO3 extractable
Available potassium	131.97 mg·kg−1	Colorimetric method by 2 mol·L−1 cold HNO3 extractable

### 2.2 Experimental design and field management

Two maize cultivars with distinct nitrogen (N) uptake characteristics, namely Zhengdan 958 (ZD958) and Qinlong 14 (QL14), were selected as experimental materials. ZD958 is a predominant commercial maize cultivar widely planted in Northwest China, characterized by a vigorous root system, superior drought tolerance, and stable grain yield under rainfed conditions; thus, it was selected as the representative local dominant cultivar. In comparison, QL14 is an emerging high-yielding cultivar increasingly popularized for spring and summer maize production in Shaanxi Province. This cultivar possesses a greater yield potential and a longer grain-filling duration, while exhibiting higher N demand during the mid and late growth stages relative to ZD958. The distinct genotypic differences in N uptake dynamics between the two cultivars provide a prerequisite for exploring the cultivar specificity of optimal urea–slow-release N fertilization regimes. In this study, five fertilization treatments were established under two N application rates (N1: 180 kg ha^-^¹; N2: 240 kg ha^-^¹), including sole urea (U), sole slow-release N fertilizer (R), and three U/R blending ratios of 2/8 (UR1), 3/7 (UR2), and 4/6 (UR3) (Garcia et al., 2018). Two non-N control treatments (CK1 for ZD958 and CK2 for QL14) were also set up for the two cultivars, respectively. All field treatments were arranged in a randomized complete block design with three biological replications.

Maize was sown at a planting density of 55,000 plants·ha^−1^, with a row spacing of 60 cm and plant spacing of 30 cm, consistent with local conventional agronomic management. Each experimental plot covered an area of 21 m², consisting of five rows with a row length of 7 m. The N fertilizers adopted in this experiment included conventional urea (U, N ≥ 46%) and commercial sulfur-coated slow-release N fertilizer (R, N ≥ 29%, valid release period of approximately three months; Stanley Commercial and Trading Co., Ltd., Shandong, China). The sulfur-coated structure of the slow-release fertilizer can block water penetration through the coating layer, thereby delaying the dissolution and release of soil N. According to the manufacturer’s specifications, the fertilizer exhibited an approximately linear cumulative N release pattern in static water at 25 °C over a 90-day period. Its N release rate was highly responsive to soil temperature: release intensity increased with rising temperature within the range of 15–35 °C, whereas soil moisture above 60% field capacity exerted negligible effects on nutrient release. Calcium superphosphate (P_2_O_5_ ≥ 16%) and potassium sulfate (K_2_O ≥ 50%) were used as phosphorus and potassium fertilizers, respectively. The basal application rates were 120 kg·ha^-^¹ for P_2_O_5_ and 60·kg ha^-^¹ for K_2_O, which were uniformly incorporated into the soil before sowing. For the sole U treatment, 30% of the total N was applied as basal fertilizer prior to sowing, and the remaining 70% was top-dressed at the jointing stage. In contrast, the R and all UR blended treatments were implemented via one-time basal fertilization, with fertilizers evenly incorporated into the 0–15 cm topsoil layer during sowing. All fertilizers were applied via furrow placement. The previous crop at the experimental field was winter wheat. During the wheat growing season, N was applied at 160 kg·ha^−1^. Wheat fields were irrigated once (30 mm) during the overwintering period, and no additional irrigation was applied thereafter. Field management was uniform across plots prior to maize planting to minimize variation in soil conditions.

### 2.3 Sampling and measurements

#### 2.3.1 NH_3_ volatilization

Soil ammonia (NH_3_) volatilization was measured using the venting method (Wang et al., 2002; Yang et al., 2018). This method presents high accuracy and precision, with a validated recovery rate of 99.51% and a coefficient of variation of 0.77%. To ensure reliable data quality, unfertilized blank control plots were set to eliminate the interference of atmospheric background ammonia, and each treatment was established with three biological replications. A PVC pipe (12 cm height, 15 cm diameter) was inserted into the topsoil to a depth of 2 cm as a volatilization collection chamber. Two circular sponges (16 cm in diameter, 2 cm in thickness) were fully soaked in 15 mL of glycerol phosphate absorption solution (prepared by diluting a mixture of 50 mL phosphoric acid + 40 mL glycerol to a constant volume of 1000 mL) and sequentially placed inside each PVC chamber. The lower sponge was fixed 5 cm above the soil surface, while the upper sponge was aligned with the top edge of the chamber. Continuous NH_3_ volatilization sampling was initiated immediately after fertilization. The lower sponge was collected daily at 8:00 a.m. to determine daily ammonia emissions. The NH_3_ adsorbed by the glycerophosphate-moistened sponge was immediately extracted with 300 mL of 1 mol·L^−1^ KCl solution and then oscillated for 1 hour. During the first week after fertilization, soil NH_3_ volatilization was measured once per day. In the second and third weeks, the sampling interval was adjusted to 1–3 days according to real-time volatilization intensity. Subsequently, the sampling interval was extended to every seven days until crop harvest. The extracted NH_4_^+^ content was determined using a continuous-flow autoanalyzer (Auto Analyzer–III, SEAL Company, Germany). Daily NH_3_ volatilization flux and cumulative NH_3_ volatilization (Equations 1, 2) were calculated following the empirical formula reported by (Chen et al., 2026):

(1) $$\text{F}\left(\text{kg}\cdot \text{N}\cdot {\text{ha}}^{-1}\cdot {\text{d}}^{-1}\right)=\frac{\text{M}}{\text{A}\times \text{D}}\times 10^{-2}$$

(2) $${\text{F}}_{\text{s}}\left(\text{kg}\cdot \text{N}\cdot {\text{ha}}^{-1}\right)=\sum \text{F}\times \text{t}$$

where *F* is the daily NH_3_ volatilization flux (kg·N·ha^−1^·d^−1^), *M* is the average NH_3_-N amount measured by a single ventilation device (mg), *A* is the cross-sectional area of the capture device (m^2^), *D* is the time of each continuous capture (d), *Fs* represents the cumulative NH_3_ volatilization over a given period (kg·N·ha^−1^), and *t* is the corresponding time period (h).

#### 2.3.2 Soil NO_3_^-^–N residue

Soil samples were collected at maize maturity to determine soil residual nitrate N (NO_3_^-^–N). For each plot, three sampling points per plot were taken between two maize plants using a soil auger, with a sampling depth of 0–120 cm. The soil profiles were evenly divided into six layers at 20-cm intervals. The fresh soil samples were air-dried, ground, and sieved through a 0.5 mm mesh screen, followed by extraction with 2 mol·L^−1^ KCl solution. Soil NO_3_^-^–N concentration was analyzed using a continuous flow analyzer (Auto Analyzer–III, SEAL Company, Germany). The cumulative accumulation of soil NO_3_^-^–N (Equation 3) throughout the 0–120 cm soil profile was calculated according to the method described by Zheng et al. (2020).

(3) $$\text{M}\left(\text{kg}\cdot {\text{ha}}^{-1}\right)=0.1\times \text{C}\times \gamma \times \text{H}$$

where *M* is the accumulated amount of soil NO_3_^-^–N (kg·ha^−1^), *C* is the content of soil NO_3_^-^–N (mg·kg^−1^), *γ* is the dry bulk density (g·cm^−3^), *H* is the soil layer thickness (cm), and 0.1 is the conversion coefficient.

#### 2.3.3 Dry matter accumulation and leaf area index

At each key maize growth stage, five uniform plants were randomly sampled from the central area of each plot to avoid marginal effects. The sampled whole plants were dissected manually into different organs (leaves, stems, and cobs) using sterilized scissors and knives. The samples were first oven-dried at 105 °C for 30 min, followed by drying at 75 °C to constant weight. Dry weights were then measured using an electronic balance.

The length (*L*) and maximum width (*W*) of each leaf from the sampled plants were measured individually (Equation 4). Leaf area was estimated using the equation:

(4) LAI=L×W×0.75

where 0.75 is the correction coefficient for maize leaves. The leaf area per plant was obtained by summing the area of all leaves. *LAI* was then calculated by multiplying the leaf area per plant by planting density.

#### 2.3.4 Photosynthesis rate

Three maize plant samples were randomly taken from each plot, and the *P*_n_ was measured using a LI-6400 photosynthesis system (LI-COR, Lincoln, NE, USA). Photosynthetically active radiation (PAR) set at 1200 μmol·m^−2^·s^−1^, reference CO_2_ concentration at 400 μmol·mol^−1^, leaf chamber temperature maintained at 25–30 °C, and relative humidity at 60–70%.

#### 2.3.5 Chlorophyll content

Three plants were randomly sampled from each plot. Fresh leaf samples (0.1 g) were extracted with 96% ethanol, and chl a and b contents were measured at 665 nm and 649 nm using a Genesys 10 UV spectrophotometer (Thermo Electron Corporation, Madison, WI, USA). Chl content was calculated as the sum of chl a and b.

#### 2.3.6 Grain yield

After physiological maturity and harvest, all maize plants within each plot were collected and air-dried to measure grain yield. The final grain yield of each plot was calculated and standardized to a 14% moisture content to unify data calibration and ensure comparability across treatments.

#### 2.3.7 N uptake and NUE

Five maize plants were sampled from different plots during critical growth stages. Samples were oven-dried at 105 °C for 0.5 h, then at 75 °C to a constant weight. The dried samples were ground, sieved (0.5mm mesh), and digested with H_2_SO_4_-H_2_O_2_. Total N content was determined using the Kjeldahl method. N uptake, vegetative organs N remobilization (VNR), N remobilization efficiency (NRE) and N use efficiency (NUE) (Equations 5–8) were calculated as follows (Chen et al., 2014; Ren et al., 2026):

(5) $$\text{N uptake}\left(\text{kg}\cdot {\text{ha}}^{-1}\right)=\text{NC}\times \text{DM}$$

(6) $$\text{VNR}\left(\text{kg}\cdot {\text{ha}}^{-1}\right)=\text{VO}\times \frac{\left(\text{NCSS}-\text{NCMS}\right)}{100}$$

(7) $$\text{NRE}\left(\%\right)=\frac{\text{VNR}}{\text{VONCS}}\times 100$$

(8) $$\text{NUE}\left(\text{kg}\cdot {\text{kg}}^{-1}\right)=\frac{\text{GY}}{\text{TNP}}$$

where, *NC* is the N content (%). *DM* is the dry matter (kg·ha^−1^). *VO* is the vegetative organs (kg·ha^−1^). *NCSS* is the N content at silking stage (%). *NCMS* is the N content at maturity stage (%). *VONCS* is the Vegetative organs N content at silking (kg·ha^−1^). *TNP* is the amount of N accumulated by above-ground plants (kg·ha^−1^).

#### 2.3.8 Comprehensive evaluation method based on entropy-weight TOPSIS method

Entropy-weight TOPSIS is widely applied in the multi-criteria evaluation and selection of agricultural management strategies (Zhao et al., 2025). In this study, to comprehensively evaluate the yield and quality of two maize cultivars, the entropy-weight TOPSIS method was employed. Fourteen indicators were selected, including grain yield, NUE, NH_3_ volatilization, soil NO_3_^-^–N, VNS (vegetative parts N uptake at silking); VNM (vegetative parts N uptake at maturity); TNM (total N uptake at maturity); PSN(Post-silking N uptake); VNR (Vegetative parts N remobilization); NRE (N remobilization efficiency); DMA (dry matter accumulation), LAI (leaf area index), Chl (chlorophyll content), and *P*_n_ (net photosynthetic rate), which cover the key factors of crop productivity, N utilization and N loss, are the core of this study (Liu et al., 2021). The specific steps of this method are as follows:

1. Data Standardization.

Assume there are 11 treatments and 14 evaluation indicators, forming the original data matrix $\text{R}={\left(r_{ij}\right)}_{11\times 14}$ (Equation 9). Normalization is performed according to the direction of each indicator (positive or negative):.

(9) $$y_{ij}=\{\begin{array}{l} \frac{r_{ij}-\min\left(r_{j}\right)}{\max\left(r_{j}\right)-\min\left(r_{j}\right)},\text{ if it is a positive indicator}\\ \frac{\max\left(r_{j}\right)-r_{ij}}{\max\left(r_{j}\right)-\min\left(r_{j}\right)},\text{ if it is a negative indicator} \end{array}$$

Obtain the standardized matrix $\text{Y}={\left({\text{y}}_{\text{ij}}\right)}_{11\times 14}$. 2. Determining weights using the entropy weight method (Equations 10–12).

Calculate the information entropy *e_j_*and weight *w_j_* of the j^th^ indicator:

(10) $${\text{p}}_{\text{ij}}=\frac{{\text{y}}_{\text{ij}}}{\sum_{\text{i}=1}^{11}{\text{y}}_{\text{ij}}}$$

(11) $${\text{e}}_{\text{j}}=–\frac{1}{\ln11}\sum_{\text{i}=1}^{11}{\text{p}}_{\text{ij }}\ln{\text{p}}_{\text{ij}}\;({\text{When }p}_{\text{ij}}=0{\text{, it is stipulated that }p}_{\text{ij}}\ln{\text{p}}_{\text{ij}}=0)$$

(12) $${\text{w}}_{\text{j}}=\frac{1-{\text{e}}_{\text{j}}}{\sum_{\text{i}=1}^{14}\left(1-{\text{e}}_{\text{j}}\right)}$$

3. Calculating closeness degree using the TOPSIS method (Equations 13–15).

Construct the weighted normalized matrix $\text{V}={\left({\text{v}}_{\text{ij}}\right)}_{11\times 14}$, where ${\text{v}}_{\text{ij}}={\text{w}}_{\text{j}}\times {\text{y}}_{\text{ij}}$. Determine the positive ideal solution ${\text{Z}}^{+}=({\text{Z}}_{1}^{+},{\text{Z}}_{2}^{+},\ldots ,{\text{Z}}_{14}^{+})$ and the negative ideal solution ${\text{Z}}^{-}=({\text{Z}}_{1}^{-},{\text{Z}}_{2}^{-},\ldots ,Z_{14}^{-})$, where:

(13) $${\text{Z}}_{\text{j}}^{+}=\max\left({\text{v}}_{1\text{j}},{\text{v}}_{2\text{j}},\ldots \ldots ,{\text{v}}_{11\text{j}}\right),{\text{Z}}_{j}^{-}=\min\left({\text{v}}_{1\text{j}},{\text{v}}_{2\text{j}},\ldots \ldots ,{\text{v}}_{11\text{j}}\right)$$

Calculate the Euclidean distances between each evaluation object and the positive and negative ideal solutions:

(14) $$D_{i}^{+}=\sqrt{\sum_{\text{j}=1}^{14}{\left(v_{ij}-Z_{j}^{+}\right)}^{2}},D_{i}^{-}=\sqrt{\sum_{j=1}^{14}{\left(v_{ij}-Z_{j}^{-}\right)}^{2}}$$

Then calculate the relative closeness degree of each object:

(15) $$C_{i}=\frac{D_{i}^{-}}{D_{i}^{+}+D_{i}^{-}},C_{i}\in \left[0,1\right]$$

Where $D_{i}^{+}$ and $D_{i}^{-}$ represent the distances from the *i^th^* treatment to the positive and negative ideal solutions, respectively; *C_i_* represents its relative closeness degree. The larger the *C_i_* value, the better the comprehensive performance of the treatment.

### 2.4 Statistical analysis

Data processing was performed using Microsoft Excel 2019. Outliers were identified using the boxplot method (1.5×IQR criterion), and none were found. Normality was assessed using the Shapiro–Wilk test. Variables satisfying normality were analyzed using one-way ANOVA, while the non-parametric Mann-Whitney U test was applied to the one variable that did not meet the normality assumption. Two-way ANOVA was performed using SPSS 23.0 on the indicator data for each cultivar separately, with N fertilizer type and N application rate as fixed factors, and their interaction effect was examined. Subsequently, Tukey’s HSD test was used to examine differences among all treatment combinations of N fertilizer type and N application rate at a significance level of *p* = 0.05. Graphs and correlation analyses were generated using Origin 2024, and Mantel tests were performed using the Chiplot online platform (https://www.chiplot.online/). The entropy-weighted TOPSIS method was applied to comprehensively evaluate and rank the combinations of fertilizer type and N application rate based on 14 indicators for each cultivar separately, in order to identify the optimal treatment for the comprehensive impact on maize grain yield and quality.

## 3 Results

### 3.1 NH_3_ volatilization and soil NO_3_^-^–N residue

#### 3.1.1 Dynamics of NH_3_ volatilization

**Figure 2:**
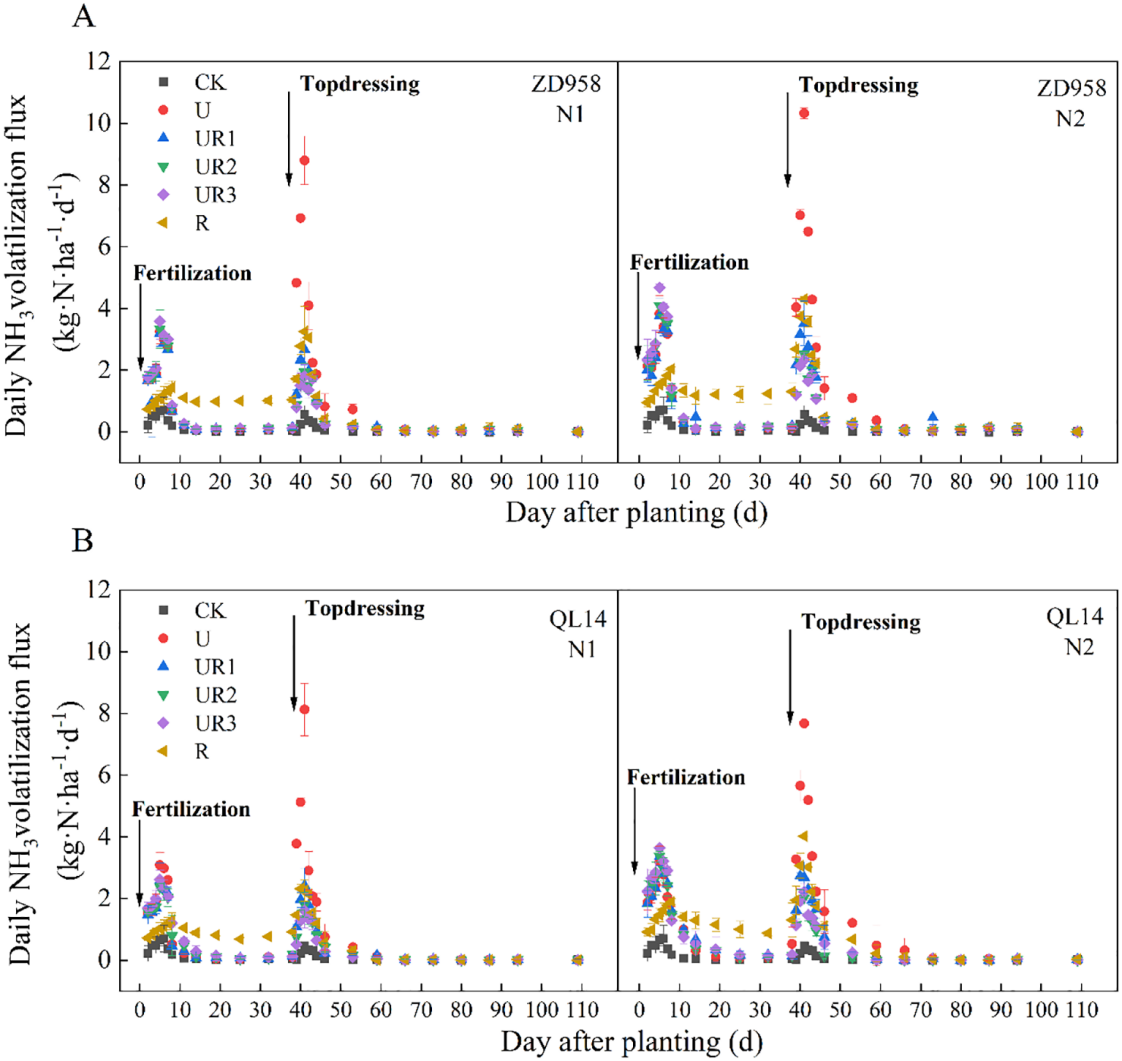
Daily NH_3_ volatilization flux of ZD958 and QL14 under different N application and fertilizer types. **(A)** Daily NH_3_ volatilization flux of ZD958. **(B)** Daily NH_3_ volatilization flux of QL14. U: urea. R: slow-release N fertilizer. UR1, UR2, and UR3: U blended with R at a ratio of 2/8, 3/7, and 4/6. N1: 180 kg·ha^−1^. N2: 240 kg·ha^−1^. Error bars represent the mean ± standard deviation (n = 3). During the first week after fertilization, soil NH_3_ volatilization was measured once per day. In the second and third weeks, the sampling interval was adjusted to 1–3 days according to real-time volatilization intensity. Subsequently, the sampling interval was extended to every seven days until crop harvest.

The daily NH_3_ volatilization flux of various treatments generally exhibited a similar trend of first increased and then decreased (**Figure 2**). The daily NH_3_ volatilization flux was higher in the first week after fertilization, then gradually declined and remained at a relatively low level. Across treatments, the daily NH_3_ volatilization flux under N2 was higher than that under N1, while CK remained at a relatively low level throughout the maize growing period. During the basal fertilization period, U and UR showed a pronounced increase in daily NH_3_ volatilization flux after U application, whereas R exhibited only a moderate increase. Compared with R, the daily NH_3_ volatilization flux under U and UR was 54.95–57.72% (N1) and 51.18–54.93% (N2) higher for ZD958, and 38.96–50.83% (N1) and 45.34–51.28% (N2) higher for QL14. Among UR treatments, the daily NH_3_ volatilization flux increased with the proportion of U, following the order UR3 > UR2 > UR1. Following top-dressing of U, an obvious peak in daily NH_3_ volatilization flux was observed, with daily NH_3_ volatilization flux following the order U > R > UR1 > UR2 > UR3 > CK. The daily NH_3_ volatilization flux of U was 144.27–210.22% (ZD958) and 209.44–247.66% (QL14) higher than that of UR, respectively. Specifically, the UN2 treatment recorded the highest daily NH_3_ volatilization flux, which were 139.42–159.40% and 93.27–154.35% greater than those in other treatments, respectively. In addition, the peak range daily NH_3_ volatilization flux of ZD958 was 1.80-10.33 kg·N·ha^−1^·d^−1^, which was higher than that of QL14.

#### 3.1.2 Cumulative NH_3_ volatilization

**Figure 3:**
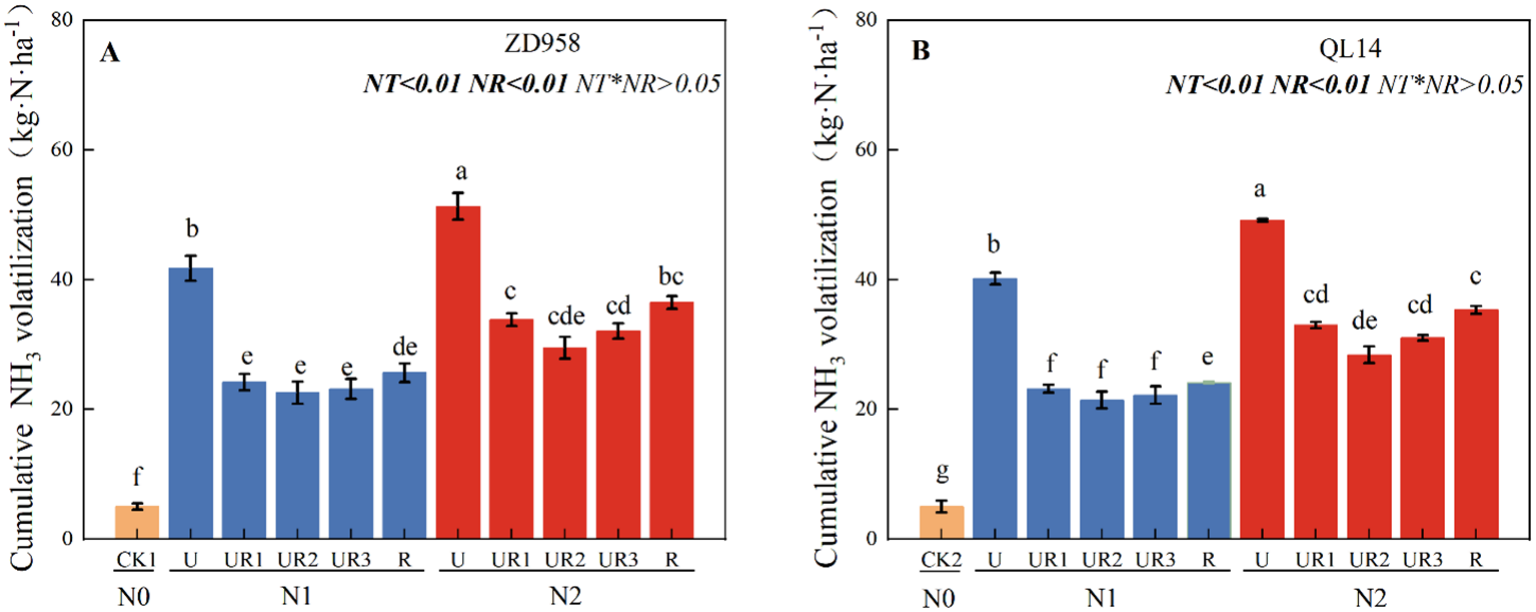
Cumulative NH_3_ volatilization of ZD958 and QL14 under different N application and fertilizer types. **(A)** NH_3_ for ZD958. **(B)** NH_3_ for QL14. U: urea. R: slow-release N fertilizer. UR1, UR2, and UR3: U blended with R at a ratio of 2/8, 3/7, and 4/6. N1: 180 kg·ha^−1^. N2: 240 kg·ha^−1^. NT: N fertilizer type. NR: N application rate. Different lowercase letters indicate significant differences (*p* < 0.05) in sample contents among different combinations of N fertilizer type and N application rate. Error bars represent the mean ± standard deviation (n = 3).

N fertilizer type and N application rate had a very significant effect on the cumulative NH_3_ volatilization of the two cultivars (*p* < 0.01), whereas their interaction had had no significant effect on the cumulative NH_3_ volatilization (*p* > 0.05). For both ZD958 and QL14, cumulative NH_3_-N increased with N application rate (**Figure 3**), with NH_3_-N under N2 being 23.63% and 22.39% higher than under N1, respectively. Across different N fertilizer types, cumulative NH_3_-N followed the order U > R > UR1 > UR3 > UR2. The UN2 treatment achieved the maximum cumulative NH_3_-N values, which were 49.94-75.55% (ZD958), and 39.12-73.10% (QL14) greater than other treatments. Cumulative NH_3_-N was lower under UR blends than under U and R. The Cumulative NH_3_ volatilization of UR2N1 treatment was 1.97–52.45% (ZD958) and 4.39–53.62% (QL14) lower than that of other treatments, respectively. Additionally, Under the same N application rate, cumulative NH_3_-N of ZD958 was 16.84% (N1) and 12.11% (N2) higher than that of QL14.

#### 3.1.3 Soil NO_3_^-^–N residue

**Figure 4:**
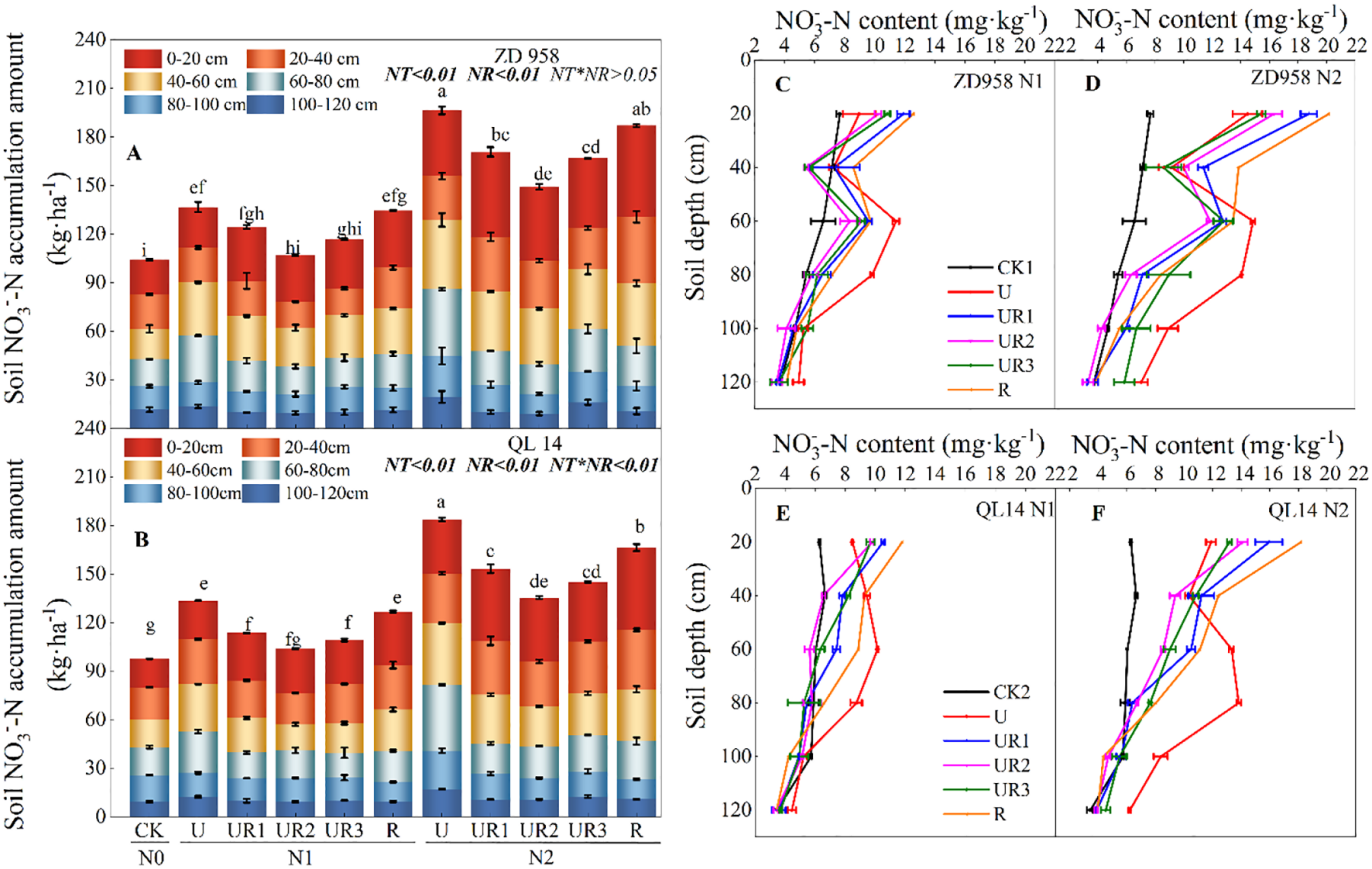
Soil NO_3_^-^–N residue in the 0–120 cm soil layer of ZD958 and QL14 under different N application and fertilizer types. (**A, B)** NO_3_^-^–N accumulation in the 0–120 cm soil layer of ZD958 and QL14. **(C, D)** NO_3_^-^–N distribution in the 0–120 cm soil of ZD958. **(E, F)** NO_3_^-^–N distribution in the 0–120 cm soil of QL14. U, urea. R, slow-release N fertilizer. UR1, UR2, and UR3: U blended with R at a ratio of 2/8, 3/7, and 4/6. N1, 180 kg·ha^−1^. N2, 240 kg·ha^−1^. NT, N fertilizer type. NR, N application rate. Different lowercase letters indicate significant differences (*p* < 0.05) in sample contents among different combinations of N fertilizer type and N application rate. Error bars represent the mean ± standard deviation (n = 3).

N fertilizer type and N application rate had a very significant effect on the soil NO_3_^-^–N accumulation of the two cultivars (*p* < 0.01), and their interaction had a significant effect on QL14 (*p* < 0.01). N fertilization significantly in-creased the accumulation of soil NO_3_^-^–N residue (**Figures 4A, B**). The soil NO_3_^-^–N accumulation in the 0–120 cm layer under N2 was 40.50% (ZD958) and 33.57% (QL14) higher than that under N1, respectively. Across different N fertilizer types, the NO_3_^-^–N residue in the 0–120 cm soil layer for both cultivars followed the order U > R > UR1 > UR3 > UR2. Compared with U, soil NO_3_^-^–N residue was 19.70% lower under UR blends and 3.60% lower under R for ZD958, and 25.15% and 8.27% lower for QL14, respectively. The lowest NO_3_^-^–N residue in the 0–120 cm layer was observed under UR2, which was 6.30–11.55% (ZD958) and 10.76–15.23% (QL14) lower than under other treatments. Additionally, under the same N fertilizer treatment, the soil NO_3_^-^–N residue of QL14 after maize harvest was 5.00–11.53% lower than that of ZD958. Under the same N application rate, the soil NO_3_^-^–N residue of QL14 after maize harvest was 2.34–9.47% (N1) and 6.93–14.98% (N2) lower than that of ZD958.

The vertical distribution of soil NO_3_^-^–N content varied markedly among N fertilizer types (**Figures 4C–F**). Under the R and blended treatments (UR), NO_3_^-^–N mainly accumulated in the 0–60 cm layer, with UR exhibiting a relatively stable distribution throughout the 0–120 cm profile. In the surface layer (0–40 cm), soil NO_3_^-^–N content followed the order R > UR > U. Compared with R, NO_3_^-^–N content under UR was 27.21–37.03% for ZD958 and 19.50–30.33% for QL14. In the deeper layer (60–120 cm), NO_3_^-^–N content followed the order U > R > UR. Compared with R and UR, NO_3_^-^–N content under U was 10.72–38.93% and 26.10–32.78% higher for ZD958, and3.96–6.73% and 17.73–27.83% higher for QL14, respectively. Additionally, observing the soil depth distribution diagram reveals that the soil NO_3_^-^–N content of ZD958 was significantly higher than that of QL14 under both N application rates. Specifically, the soil NO_3_^-^–N content of ZD958 in the 0–120 cm soil layer was 5.64% (N1) and 11.3% (N2) higher than that of QL14.

### 3.2 N remobilization after silking stage

The cultivars had significant effect on N uptake of vegetative organs at silking (VNS) and maturity stages (VNM) (*p* < 0.05) (**Table 2**). N fertilizer type and application rate significantly affected VNS, VNM, total N uptake at maturity (TNM), post-silking N uptake (PSN), vegetative N remobilization (VNR), and N remobilization efficiency (NRE) (*p* < 0.05). The interactions of cultivar and N fertilizer type or N application rate had no significant effects on these six parameters (*p*>0.05), while the interactions of N fertilizer type and N application rate had significant effects on VNS, VNM, TNM and NRE (*p* < 0.01). Under N1, all N uptake and remobilization parameters were consistently higher than under N2. Among fertilizer types, urea (U) exhibited the lowest values for these parameters across all treatments. All N uptake and remobilization parameters in R were 5.34–16.90%, 3.22–15.14%, 5.40–13.99%, 9.25–20.12% and 2.72–5.84% higher than those in UR, respectively. For UR, these six parameters of N uptake and remobilization increased with the increasing ratio of U and R, and following the order of UR2 > UR3 > UR1. VNS, VNM, TNM and VNR in UR2 were 3.84–10.97%, 5.58–11.55%, 4.58–8.15% and 0.94–9.95% higher than other treatments. QL14 generally showed higher N uptake and remobilization parameters than ZD958, with the difference being most pronounced for VNR (5.04%). PSN was comparable between the two cultivars, with only a negligible difference (0.06%).

**Table 2:** Post-silking N remobilization of ZD958 and QL14 under N application rate and N fertilizer type.

Treatments	VNS (kg ha^−1^)	VNM (kg ha^−1^)	TNM (kg ha^−1^)	PSN (kg ha^−1^)		VNR (kg ha^−1^)		NRE (%)
N fertilizer type (NT)								
CK	62.12 ± 0.79 e	45.60 ± 0.31 e	70.66 ± 1.03 d	8.53 ± 0.30 c	16.52 ± 0.55 c		26.58 ± 0.57 b	
U	94.82 ± 3.75 d	60.53 ± 2.11 d	120.90 ± 3.94 c	26.07 ± 1.82 b	34.29 ± 2.08 b		36.02 ± 1.12 a	
UR1	114.41 ± 2.50 bc	72.49 ± 0.97 bc	150.60 ± 3.14 ab	36.19 ± 1.30 a	41.93 ± 1.67 ab		36.55 ± 0.69 a	
UR2	126.96 ± 1.45 a	80.86 ± 1.19 a	162.88 ± 2.32 a	35.93 ± 0.98 a	46.10 ± 2.42 a		36.20 ± 1.52 a	
UR3	122.27 ± 1.27 ab	76.59 ± 0.66 ab	155.75 ± 1.61 a	33.48 ± 1.46 a	45.67 ± 1.60 a		37.30 ± 0.97 a	
R	108.61 ± 2.78 c	70.23 ± 1.35 c	142.89 ± 2.84 b	34.29 ± 1.03 a	38.38 ± 1.71 ab		35.24 ± 0.84 a	
N application rate (NR)								
N0	62.12 ± 0.79 b	45.60 ± 0.31 b	70.66 ± 1.03 b	8.53 ± 0.30 b	16.52 ± 0.55 c		26.58 ± 0.57 c	
N1	118.74 ± 2.23 a	73.49 ± 0.97 a	153.62 ± 2.94 a	34.88 ± 1.15 a	45.25 ± 1.40 a		37.95 ± 0.56 a	
N2	108.09 ± 3.16 a	70.79 ± 2.27 a	139.59 ± 3.76 a	31.50 ± 1.08 a	37.29 ± 1.09 b		34.57 ± 0.52 b	
Cultivar (C)								
ZD958	107.28 ± 4.26 a	69.22 ± 2.28 a	138.24 ± 5.83 a	30.96 ± 1.85 a	38.06 ± 2.15 a		34.91 ± 0.89 a	
QL14	110.22 ± 4.15 a	70.24 ± 2.34 a	141.16 ± 5.86 a	30.94 ± 1.91 a	39.98 ± 2.02 a		35.86 ± 0.81 a	
ANOVA								
C	< 0.05	< 0.05	**< 0.01**	> 0.05	> 0.05		> 0.05	
NT	**< 0.01**	**< 0.01**	**< 0.01**	**< 0.01**	**< 0.01**		> 0.05	
NR	**< 0.01**	**< 0.01**	**< 0.01**	**< 0.01**	**< 0.01**		**< 0.01**	
NT*C	> 0.05	> 0.05	> 0.05	> 0.05	> 0.05		> 0.05	
NR*C	> 0.05	> 0.05	> 0.05	> 0.05	> 0.05		> 0.05	
NT*NR	**< 0.01**	**< 0.01**	**< 0.01**	> 0.05	> 0.05		< 0.05	
NT*NR*C	> 0.05	> 0.05	> 0.05	> 0.05	> 0.05		> 0.05	

U, urea; R, slow-release N fertilizer; UR1, UR2, and UR3, U blended with R at a ratio of 2/8, 3/7, and 4/6; N1, 180 kg·ha^−1^; N2, 240 kg·ha^−1^; NT, N fertilizer type; NR, N application rate; C, Cultivars; VNS, vegetative parts N uptake at silking; VNM, vegetative parts N uptake at maturity; TNM, total N uptake at maturity; PSN, Post-silking N uptake; VNR, Vegetative parts N remobilization; NRE, N remobilization efficiency. Different lowercase letters indicate significant differences (*p* < 0.05) in sample contents among different combinations of N fertilizer type, N application rate and cultivar. Error bars represent the mean ± standard deviation (n = 3). Bold values indicate highly significant differences at *p* < 0.01.

### 3.3 Dry matter accumulation and leaf area index

**Figure 5:**
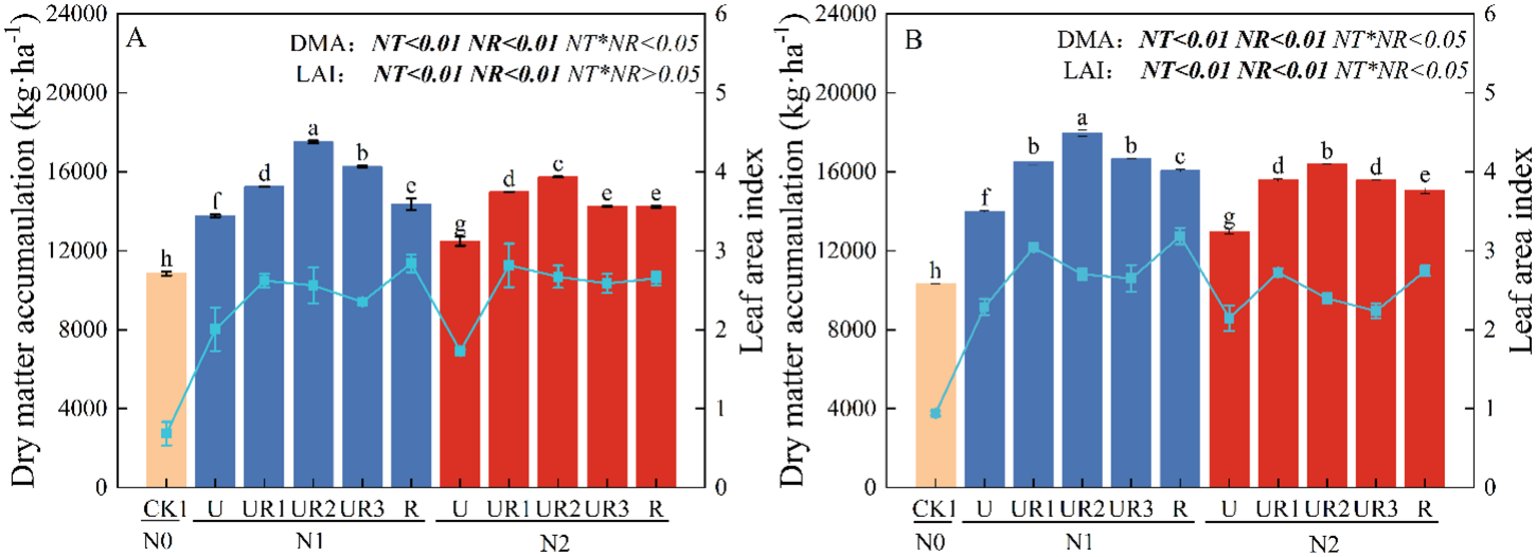
Dry matter accumulation (DMA) and leaf area index (LAI) of ZD958 and QL14 at different growth stages of maize under different N application and fertilizer types. **(A)** DMA and LAI for ZD958. **(B)** DMA and LAI for QL14. U, urea. R, slow-release N fertilizer. UR1, UR2, and UR3: U blended with R at a ratio of 2/8, 3/7, and 4/6. N1: 180 kg·ha^−1^. N2: 240 kg·ha^−1^. NT, N fertilizer type. NR, N application rate. Different lowercase letters indicate significant differences (*p* < 0.05) in sample contents among different combinations of N fertilizer type and N application rate. Error bars represent the mean ± standard deviation (n = 3). The bar chart represents DMA, while the line chart with dots represents LAI.

N fertilizer type and N application rate had significant effects on the dry matter accumulation (DMA) and leaf area index (LAI) of ZD958 and QL14 at maturity stage (*p* < 0.01). Their interaction had a significant effect on the DMA of both maize cultivars and on the LAI of QL14 (*p* < 0.05), but showed no significant effect on the LAI of ZD958 (*p* > 0.05) (**Figure 5**). DMA under all fertilization treatments was higher than that under CK. Across different N fertilizer types, DMA followed the order of UR2 > UR1 > UR3 > R > U. Compared with U, blended fertilizer (UR) treatments increased DMA. Compared with R, the increases ranged 0.17–21.92% (ZD958) and 2.70–11.82% (QL14). Among UR treatments, UR2 achieved the highest DMA, which was 5.09–14.83% higher than UR1 and UR3 in ZD958, and 5.15–7.82% higher in QL14. Under different N application rates, DMA under N1 was higher than under N2. The highest DMA for both cultivars was observed under UR2N1which were 7.70–61.49% (ZD958) and 7.82–73.98% (QL14) higher than other treatments, respectively. Under the same N fertilizer type, DMA of QL14 was 2.67–8.81% higher than that of ZD958. Under the same N application rate, DMA of QL14 was higher than that of ZD958.

Under different N fertilizer types, LAI followed the order of R > UR1 > UR2 > UR3 > U. Compared with U, UR treatments increased LAI by 17.21–62.72% in ZD958 and 4.20–33.04% in QL14. Compared with R, LAI decreased by 2.62–20.85% in ZD958 and by 0.73–22.82% in QL14. Among UR treatments, UR1 achieved the highest LAI. Under different N application rates, LAI of ZD958 under N1 was lower than under N2, with a decrease of 4.30–10.00%. LAI of QL14 under N1 was higher than under N2. Overall, the highest LAI for both cultivars was observed under RN1, which was 0.89–64.16% and 4.77–48.49% higher than other treatments, respectively. Furthermore, QL14 exhibited better DMA performance than ZD958 across different N fertilizer types and N application rates. Under the same N fertilizer type, LAI of QL14 was 3.59–23.99% higher than that of ZD958. Under the same N application rate, LAI of QL14 was 5.49–16.03% (N1) and 3.59–23.99% (N2) higher than that of ZD958, respectively.

### 3.4 Net photosynthetic rate and chlorophyll content

#### 3.4.1 Net photosynthetic rate

N fertilizer type and N application rate had significant effects on the net photosynthetic rate (*P*_n_) of maize at different growth stages (*p* < 0.01) (**Table 3**). The interaction of N fertilizer type and N application rate had a significant effect on *P*_n_ of ZD958 and QL14 at jointing stage (*p* < 0.01). Across the growing season, *P*_n_ increased from the seedling stage to the jointing stage and declined thereafter, reaching its maximum at the jointing stage. The response of *P*_n_ to N rate varied with growth stage and fertilizer type. At the seedling stage, *P*_n_ of U, R, and UR under N2 was significantly higher than under N1. At the jointing stage, *P*_n_ of U under N2 was higher than under N1, whereas *P*_n_ of UR and R under N2 was significantly lower than under N1. At the silking and filling stages, *P*_n_ of U, UR, and R under N1 was significantly higher than under N2. Among the UR blends, the ranking of *P*_n_ varied by growth stage: at the seedling and silking stages, the order was UR3 > UR2 > UR1; at the jointing and filling stages, the order was UR2 > UR3 > UR1. However, UR2 was significantly lower than UR3 at the seedling stage and significantly lower than UR1 at the filling stage. At the silking stage, *P*_n_ in UR2 was 7.21–8.10% (ZD958) and 6.05–9.98% (QL14) higher than other treatments. Additionally, QL14 consistently exhibited higher *P*_n_ than ZD958 across all growth stages, fertilizer types, and N application rates.

**Table 3:** Net photosynthetic rate of ZD958 and QL14 under different N application rates and N fertilizer types.

Net photosynthetic rate (μmol·m^−2^·s^−1^)						
Cultivar	Treatment		Seeding	Jointing	Silking	Filling
ZD958	CK	N0	21.91 ± 0.99 g	30.16 ± 0.57 f	29.32 ± 1.67 d	23.64 ± 0.76 c
	U	N1	28.07 ± 0.32 de	38.79 ± 0.44 bcd	36.40 ± 0.85 ab	25.04 ± 1.69 bc
		N2	32.00 ± 0.84 bc	44.23 ± 0.74 a	33.92 ± 1.65 bcd	24.33 ± 0.58 c
	UR1	N1	25.51 ± 0.90 ef	38.51 ± 1.15 bcd	37.08 ± 1 ab	31.96 ± 0.56 a
		N2	30.39 ± 0.96 cd	35.35 ± 0.22 de	34.98 ± 0.45 abc	27.78 ± 0.36 abc
	UR2	N1	29.83 ± 0.54 cd	41.6 ± 1.29 ab	40.09 ± 1.08 a	30.19 ± 1.55 ab
		N2	34.05 ± 0.10 ab	38.61 ± 0.44 bcd	36.55 ± 0.36 ab	26.94 ± 0.10 abc
	UR3	N1	30.47 ± 0.44 cd	40.57 ± 1.10 abc	37.39 ± 0.07 ab	27.67 ± 1.26 abc
		N2	35.60 ± 0.18 a	36.96 ± 0.64 cd	35.87 ± 1.05 abc	26.87 ± 0.06 abc
	R	N1	24.20 ± 0.62 fg	35.19 ± 0.01 de	34.23 ± 0.58 bcd	32.45 ± 1.28 a
		N2	24.59 ± 0.10 efg	32.39 ± 0.64 ef	30.59 ± 0.49 cd	30.82 ± 1.14 a
ANOVA		NT	**< 0.01**	**< 0.01**	**< 0.01**	**< 0.01**
		NR	**< 0.01**	< 0.05	**< 0.01**	**< 0.01**
		NT*NR	**< 0.01**	**< 0.01**	> 0.05	> 0.05
QL14	CK	N0	23.51 ± 0.57 g	30.85 ± 0.46 e	30.78 ± 0.51 f	23.74 ± 0.54 d
	U	N1	30.97 ± 0.80 cde	41.95 ± 0.08 abc	37.09 ± 1.22 bcd	27.04 ± 0.42 cd
		N2	34.72 ± 0.94 abc	45.96 ± 1.03 a	34.08 ± 0.21 de	24.94 ± 0.06 d
	UR1	N1	27.34 ± 0.37 efg	40.67 ± 0.89 abc	38.49 ± 0.46 bc	33.16 ± 0.13 ab
		N2	32.29 ± 0.32 bcd	36.66 ± 0.20 cd	35.87 ± 0.78 cd	29.81 ± 0.27 bc
	UR2	N1	31.04 ± 0.32 cde	42.86 ± 0.25 ab	42.33 ± 0.29 a	33.51 ± 1.18 ab
		N2	36.33 ± 1.24 ab	40.10 ± 2.04 bcd	38.80 ± 0.23 bc	28.23 ± 1.27 cd
	UR3	N1	32.95 ± 0.39 bcd	41.81 ± 1.18 abc	39.92 ± 0.31 ab	29.70 ± 0.59 bc
		N2	37.36 ± 0.03 a	38.41 ± 0.49 bcd	37.44 ± 0.51 bc	27.74 ± 1.04 cd
	R	N1	26.28 ± 0.15 fg	37.91 ± 1.29 bcd	36.09 ± 0.25 cd	34.54 ± 0.79 a
		N2	28.67 ± 1.62 def	35.37 ± 0.48 de	32.30 ± 0.70 ef	31.45 ± 1.49 abc
ANOVA		NT	**< 0.01**	**< 0.01**	**< 0.01**	**< 0.01**
		NR	**< 0.01**	< 0.05	**< 0.01**	**< 0.01**
		NT*NR	> 0.05	**< 0.01**	> 0.05	> 0.05

U, urea; R, slow-release N fertilizer; UR1, UR2, and UR3, U blended with R at a ratio of 2/8, 3/7, and 4/6; N1, 180 kg·ha^−1^; N2, 240 kg·ha^−1^; NT, N fertilizer type; NR, N application rate. Different lowercase letters indicate significant differences (p< 0.05) in sample contents among different combinations of N fertilizer type and N application rate. Error bars represent the mean ± standard deviation (n = 3). Bold values indicate highly significant differences at *p* < 0.01.

#### 3.4.2 Chlorophyll content

**Figure 6:**
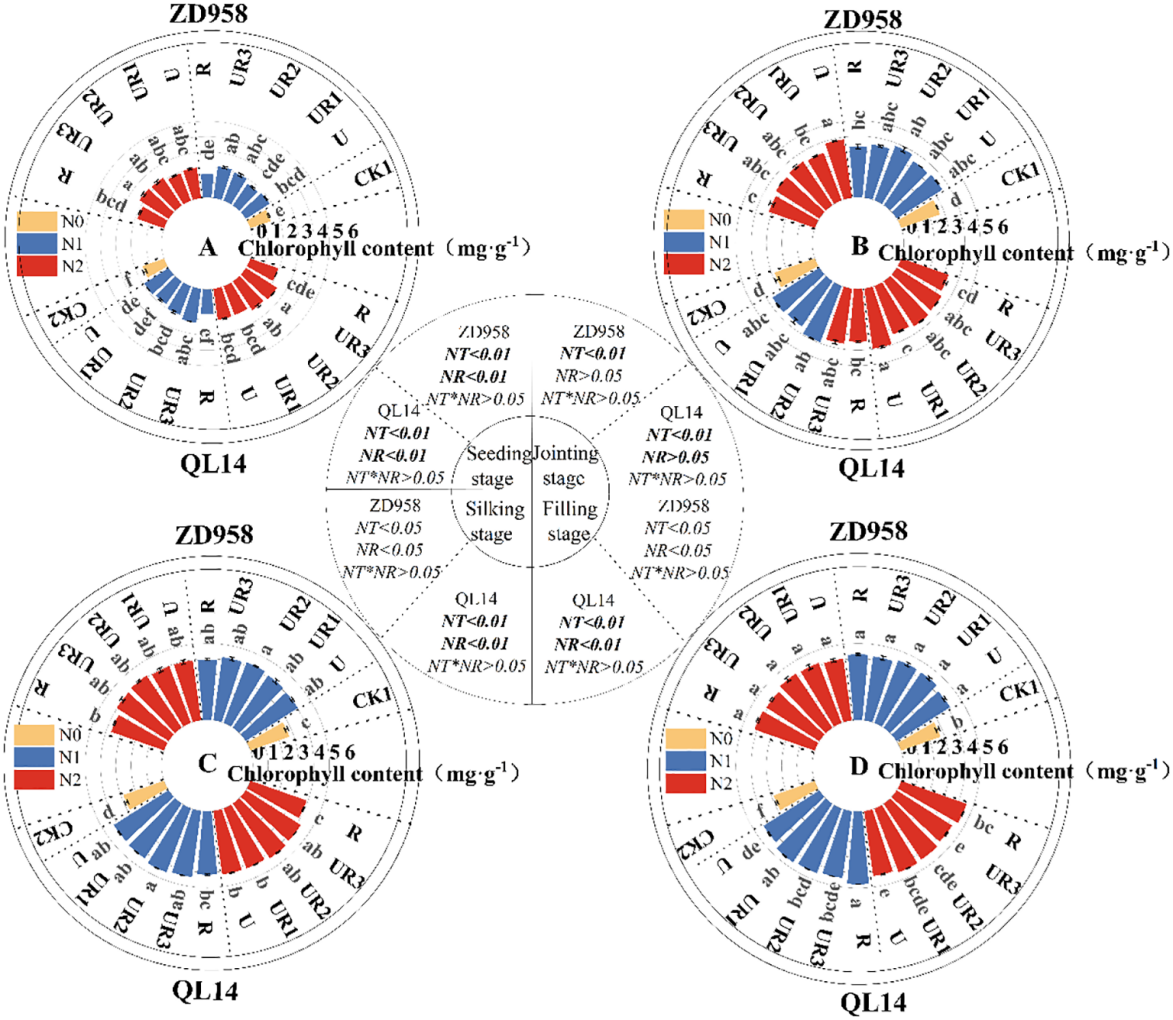
Chlorophyll content (Chl) of ZD958 and QL14 at different growth stages of maize under different N application and fertilizer types. **(A–D)** Chl at the seedling, jointing, silking and filling stage of ZD958 and QL14. U, urea. R, slow-release N fertilizer. UR1, UR2, and UR3: U blended with R at a ratio of 2/8, 3/7, and 4/6. N1: 180 kg·ha^−1^. N2: 240 kg·ha^−1^. NT, N fertilizer type. NR, N application rate. Different lowercase letters indicate significant differences (*p* < 0.05) in sample contents among different combinations of N fertilizer type and N application rate. Error bars represent the mean ± standard deviation (n = 3).

Fertilizer type and N application rate significantly affected chlorophyll content (Chl) at most growth stages (**Figure 6**). At the seedling and silking stages, both factors showed highly significant effects in the two cultivars (*p* < 0.01). Significant interactions between fertilizer type and N rate were observed at the jointing stage in QL14 (*p* < 0.05). In contrast, no significant main or interaction effects were detected at the filling stage (*p* > 0.05). Under the same N application rate, the ranking of Chl varied by growth stage. At the seedling stage, the Chl of U, R, and UR treatments under N2 was higher than that under N1. At the jointing stage under N2, the Chl of U was higher than that under N1, while that of UR and R was significantly lower than under N1. At the silking and filling stages, the Chl of U, UR, and R under N1 was significantly higher than that under N2. Among the UR blends, the ranking of Chl varied by growth stage: at the seedling stage, the order was UR3 > UR2 > UR1; at the silking, jointing, and filling stages, the order was UR2 > UR3 > UR1. In both cultivars, the Chl of UR2 was significantly higher than that of U and R during the mid-to-late growth stages. However, the Chl of UR2 was lower than that of UR3 at the seedling stage. At the silking stage, the Chl of UR2N1 was 1.68–3.78% (ZD958) and 1.76–3.70% (QL14) higher than that of UR1 and UR3, respectively. Additionally, under the same fertilizer type and N application rate, the Chl of QL14 was higher than that of ZD958.

### 3.5 Grain yield and NUE

**Figure 7:**
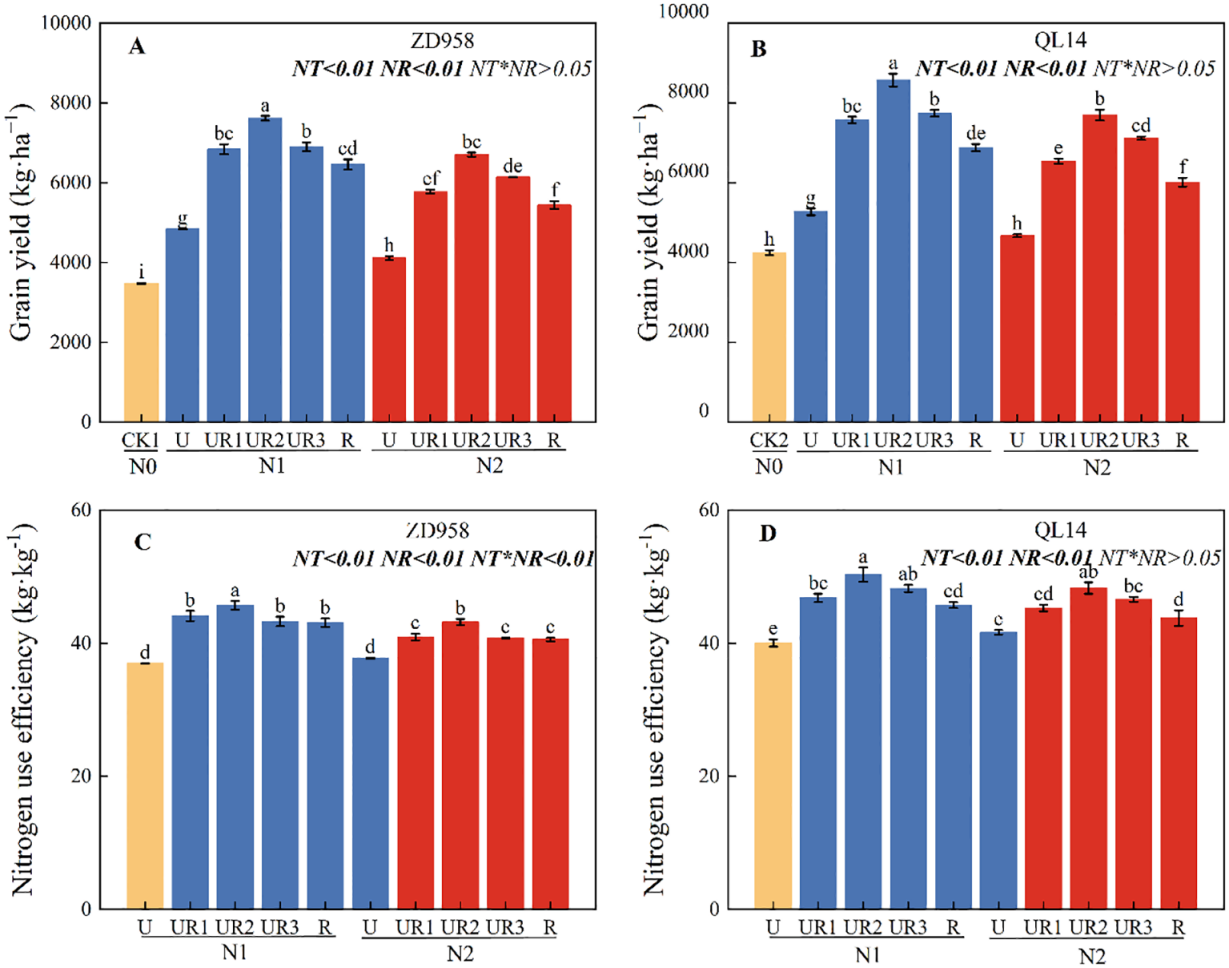
Maize yield and N use efficiency (NUE) of ZD958 and QL14 under different N application and fertilizer types. **(A)** Grain yield for ZD958. **(B)** Grain yield for QL14. **(C)** NUE for ZD958. **(D)** NUE for QL14. U, urea. R, slow-release N fertilizer. UR1, UR2, and UR3: U blended with R at a ratio of 2/8, 3/7, and 4/6. N1: 180 kg·ha^−1^. N2: 240 kg·ha^−1^. NT, N fertilizer type. NR, N application rate. Different lowercase letters indicate significant differences (*p* < 0.05) in sample contents among different combinations of N fertilizer type and N application rate. Error bars represent the mean ± standard deviation (n = 3).

N fertilization significantly increased the grain yield and N use efficiency (NUE) of ZD958 and QL14 (**Figures 7A, B**). N fertilizer type and N application rate had significant effects on the yield and NUE of the two cultivars (*p* < 0.01). The interaction between N fertilizer type and N application rate had a significant effect on the NUE of ZD958 (*p* < 0.01). For both ZD958 and QL14, grain yield under different N fertilizer types followed the order: UR2 > UR1 > UR3 > R > U. Across UR fertilizers, grain yield was 46.33–55.86% higher than that of U, and 5.59–12.46% higher than that of R, with UR2 outperforming UR3 by 5.78% (ZD958) and 9.81% (QL14). Yield was consistently higher under N1 than N2. The highest grain yield for both cultivars was observed under UR2N1, reaching 8573.14 kg·ha^−1^ for ZD958 and 7610.57 kg·ha^−1^ for QL14, which were 9.7–54.7% and 9.4–54.3% higher than other treatments, respectively. Furthermore, grain yield was consistently higher in QL14 than in ZD958 across different N fertilizer types and application rates. Under the same N fertilizer type, grain yield of QL14 was 5.95–17.40% higher than that of ZD958. Under the same N application rate, QL14 yielded 10.43-13.71% more than ZD958, respectively.

Similar trends were observed for NUE of ZD958 and QL14 (**Figures 7C, D**). Under different N fertilizer types, NUE followed the same order for both cultivars: UR2 > UR1 > UR3 > R > U. Across UR fertilizers, NUE was 0.37–22.77% higher than that of U, and 0.79–2.79% higher than that of R. In UR fertilizers, UR2 had the highest NUE, which was 2.71% (ZD958) and 4.17% (QL14) higher than UR3. NUE was consistently higher under N1 than N2. The highest NUE for both cultivars was observed under UR2N1, reaching 45.70 kg·kg^−1^ for ZD958 and 50.31 kg·kg^−1^ for QL14. Furthermore, QL14 exhibited better NUE performance than ZD958 across different N fertilizer types and application rates. NUE of QL14 was increased by 1.94–14.41% compared with ZD958. Under the same N application rate, NUE of QL14 was 1.94% and 14.41% higher than that of ZD958.

### 3.6 Correlation analysis between soil N residue, physiological growth, yield and NUE

**Figure 8:**
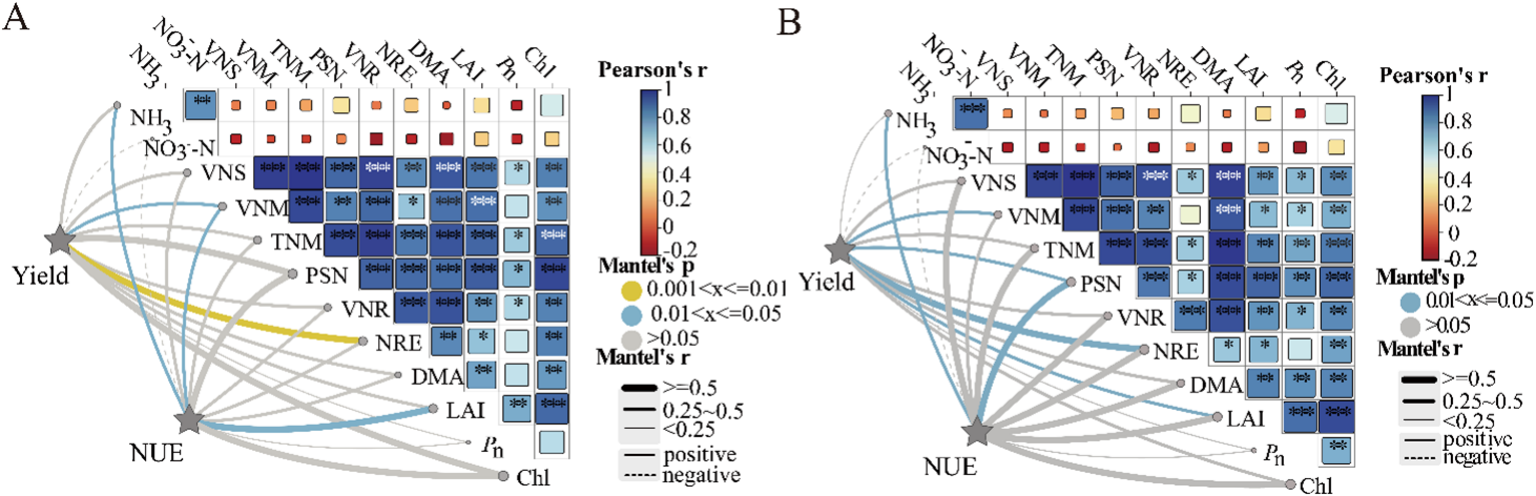
Correlation analysis between soil N residue, N remobilization after silking stage, physiological growth, yield and NUE of two maize cultivars under different fertilizer types and N application rates. **(A)** Correlation analysis of ZD958. **(B)** Correlation analysis of QL14. VNS: vegetative parts N uptake at silking. VNM: vegetative parts N uptake at maturity. TNM, total N uptake at maturity; PSN, Post-silking N uptake; VNR, Vegetative parts N remobilization; NRE, N remobilization efficiency; DMA, Dry matter accumulation; LAI, Leaf area index; *P*_n_, Net photosynthesis rate; Chl, Chlorophyll content; NUE, N use efficiency. P denotes significance. r denotes the correlation coefficients.

Mantel test and Pearson correlation analysis were used to study the relationships between soil N residue, physiological growth, yield, and NUE of the two maize cultivars (**Figure 8**). For ZD958, cumulative NH_3_ volatilization and NO_3_^-^–N residue were negatively correlated with PSN, NRE, DMA, *P*_n_, and Chl, respectively. In contrast, PSN was significantly positively correlated with NRE, DMA, *P*_n_, and Chl. Significant positive correlations were also observed among NRE, DMA, *P*_n_, and Chl. NUE was significantly correlated with LAI but weakly correlated with other traits. Yield showed a relatively weak correlation with most indicators. For QL14, cumulative NH_3_ volatilization and NO_3_^-^–N residue were significantly correlated with PSN, NRE, DMA, LAI, *P*_n_, and Chl. PSN was significantly positively correlated with NRE, DMA, *P*_n_, and Chl. Additionally, NRE and DMA were closely related to *P*_n_ and Chl. In contrast to ZD958, yield in QL14 was significantly correlated with cumulative NH_3_ volatilization, NO_3_^-^–N residue, PSN, and DMA, while NUE was significantly correlated with cumulative NH_3_ volatilization, PSN, and DMA.

### 3.7 Comprehensive evaluation of yield improvement and NUE enhancement in two maize varieties based on the TOPSIS method

Based on the entropy-weight TOPSIS comprehensive evaluation model, 14 indicators under different N fertilizer management practices were systematically analyzed for ZD958 and QL14 (**Table 4**). The results showed that the ranking trends of the comprehensive scores were highly consistent between the two varieties. For ZD958, the ranking order was: UR2N1 > UR1N1 > RN1 > UR3N1 > UR2N2 > UR1N2 > RN2 > UR3N2 > UN1 > UN2 > CK. For QL14. For QL14, the ranking order was: UR2N1 > UR1N1 > RN1 > UR3N1 > UR2N2 > UR1N2 > RN2 > UR3N2 > UN1 > UN2 > CK. For both ZD958 and QL14, the top four treatments were UR2N1, UR1N1, RN1, and UR3N1 in descending order. The UR2N1 treatment (3/7, 180 kg·ha^−1^) ranked first for both varieties, with comprehensive scores of 0.811 (ZD958) and 0.817 (QL14), respectively.

**Table 4:** Euclidean distances (*Di^+^* and *Di^-^*) of the target values for ZD958 and QL14 maize from the ideal points, and the relative closeness coefficients (Ci) for each treatment.

Evaluation object		ZD958				QL14			
N fertilizer type	N application rate	Euclidean Distance		Relative proximity Coefficient *Ci*	Rank	Euclidean Distance		Relative proximity Coefficient *Ci*	Rank
		*Di^+^*	*Di^−^*			*Di^+^*	*Di^−^*		
CK	N0	0.245	0.137	0.359	11	0.246	0.126	0.337	11
U	N1	0.165	0.142	0.461	9	0.157	0.139	0.469	9
	N2	0.214	0.129	0.376	10	0.190	0.150	0.440	10
UR1	N1	0.061	0.233	0.794	2	0.067	0.222	0.767	2
	N2	0.135	0.166	0.551	6	0.121	0.170	0.585	6
UR2	N1	0.057	0.244	0.811	1	0.054	0.240	0.817	1
	N2	0.117	0.191	0.620	5	0.109	0.191	0.637	5
UR3	N1	0.085	0.218	0.718	4	0.072	0.218	0.753	4
	N2	0.151	0.163	0.520	8	0.149	0.161	0.519	8
R	N1	0.074	0.226	0.754	3	0.068	0.223	0.767	3
	N2	0.145	0.172	0.543	7	0.137	0.162	0.543	7

*Di^+^*, positive ideal solution distance; *Di^-^*, negative ideal solution distance. U, urea. R, slow-release N fertilizer. UR1, UR2, and UR3, U blended with R at a ratio of 2/8, 3/7, and 4/6. N1, 180 kg·ha^−1^. N2, 240 kg·ha^−1^.

## 4 Discussion

### 4.1 Effects of fertilizer type and N application rate on NH_3_ volatilization

Nitrogen (N) fertilization is widely acknowledged as the primary factor accelerating soil ammonia (NH_3_) volatilization in agricultural ecosystems (Wang et al., 2018). In this study, NH_3_ volatilization increased with increasing N application rate, which is consistent with previous findings (Li et al., 2017). The cumulative NH_3_ volatilization loss under N2 was higher than that N1. This can be attributed to the rapid hydrolysis of a large amount of applied N fertilizer, leading to inorganic N in the soil that maize cannot be taken up by maize in time, thereby increasing NH_3_ emissions (He et al., 2018). Previous studies have also shown that large amounts of N fertilizer are rapidly hydrolyzed to NH_4_^+^–N by urease secreted by soil microorganisms after basal fertilizer application, increasing soil NH_4_^+^–N concentrations, which may contribute to higher NH_3_ emission rates (Mencaroni et al., 2021). In this study, daily NH_3_ volatilization flux from U, UR, and R peaked shortly after fertilization and then gradually decreased to a stable value. This can be explained by the uptake of NH_4_^+^–N by maize, while the remaining NH_4_^+^–N is either adsorbed by the soil or converted to NO_3_^–^–N via nitrification, which may have reduced and stabilized NH_3_ volatilization (Yu et al., 2019).

Cumulative NH_3_ volatilization in the R and UR treatments was lower than in U treatment. This may be due to the coating material of R blocking contact between urea inside the membrane and soil urease, thereby impeding water transport necessary for urea dissolution, slowing N release, and reducing NH_3_ volatilization (Lawrencia et al., 2021). Furthermore, R and UR treatments were applied as basal fertilizers to the 15 cm soil layer, where the NH_4_^+^–N produced by hydrolysis was more readily available for uptake by maize and soil adsorption. Therefore, the R and UR treatments may significantly reduce NH_3_ volatilization compared to topdressing urea on the soil surface. Among the UR treatments, cumulative NH_3_ volatilization followed the order UR1 > UR3 > UR2, with UR2 showing the lowest NH_3_ loss. This finding is consistent with the results of Cao et al. (2024), who reported that in a winter wheat–summer maize rotation system in the North China Plain, blending coated and common urea at a 3/7 ratio (maize) significantly reduced annual ammonia volatilization by 69.70–71.90% compared with conventional fertilization.

Several mechanisms may explain why the 3/7 ratio (UR2) was most effective in reducing NH_3_ volatilization compared with UR1 (2/8) and UR3 (4/6). First, the 3/7 ratio (UR2) likely achieved a more optimal balance between rapid N availability and sustained N supply. UR1 may have released N too slowly during the early growth stages, leading to a mismatch between N supply and crop demand at the seedling stage, while UR3 may have released N too rapidly, increasing the risk of NH_3_ loss during the peak volatilization period (Wang M. et al., 2025). In contrast, UR2 may have provided a more gradual and sustained N release pattern that better matched the temporal pattern of maize N uptake. Second, the 30% urea in UR2was sufficient to meet the immediate N demand of maize at the seedling stage, while the 70% slow-release N component sustained N availability during the mid-to-late reproductive stages, thereby reducing the accumulation of inorganic N in the soil that would otherwise be susceptible to volatilization (Garcia et al., 2018). Third, the enhanced N uptake and remobilization efficiency under UR2 reduced the substrate available for volatilization. Efficient crop N uptake creates a positive feedback loop that lowers soil residual inorganic N, thereby minimizing volatilization peaks (Gao et al., 2023). These results collectively demonstrate that optimizing the blending ratio to synchronize N supply with crop demand is the key to mitigating NH_3_ volatilization losses. The lower daily NH_3_ volatilization flux in UR compared with the R may be related to improved temporal alignment between N supply and crop N demand, potentially facilitating N uptake and reducing volatilization losses (Vo et al., 2021). These results align with previous findings showing that UR effectively sustained N uptake throughout the growing season, reducing excess soil NH_4_^+^–N and thus minimizing NH_3_ volatilization losses (Gautam et al., 2022).

Soil NH_3_ volatilization and NO_3_^–^–N residue were significantly negatively correlated with NUE in both cultivars, highlighting the importance of reducing N losses to improve NUE (Bhatt et al., 2025). NUE was also influenced by genotypic differences between the two cultivars. In the present study, cumulative NH_3_ volatilization was higher in ZD958 than in QL14. This difference may be related to variations in N uptake capacity, as N-efficient cultivars such as QL14 may absorb NH_4_^+^–N more effectively, thereby reducing the substrate available for NH_3_ volatilization (Ruswandi et al., 2022). Previous studies have reported that genotypic variation in N uptake and assimilation is associated with differences in root activity and N metabolism-related enzymes (Plett et al., 2016). Enhanced N uptake efficiency may reduce soil ammonium accumulation, which in turn could limit NH_3_ volatilization (Zheng et al., 2020). The genotypic difference in NH_3_ volatilization observed between QL14 and ZD958 is also supported by studies on variety-specific responses to controlled-release fertilizers. Ma et al. (2024) demonstrated that blending controlled-release urea with common urea achieved the highest yield and reduced greenhouse gas intensity when matched with suitable maize varieties, highlighting that cultivar selection is critical for maximizing the benefits of controlled-release blended fertilizers. Our results further show that the N-efficient cultivar QL14 not only reduced NH_3_ volatilization but also exhibited a stronger correlation network linking N uptake, photosynthetic capacity, and yield formation, suggesting that genotype-specific physiological traits should be considered when optimizing blending ratios. These mechanisms may partly explain the lower cumulative NH_3_ volatilization observed in QL14 compared with ZD958.

### 4.2 Effects of fertilizer type and N application rate on soil NO_3_^-^–N residue of two maize cultivars

Appropriate N fertilizer management has been shown to maintain high grain yield, improve NUE, and reduce N leaching (Liu et al., 2021). In this study, increasing the N application rate significantly increased the residual NO_3_^-^–N in the 0–120 cm soil layer. This is likely due to the excess N accumulating in the soil as NO_3_^-^–N when the N application rate exceeded maize uptake capacity, with subsequent leaching deeper soil layers by rainfall (Yu et al., 2019). In the 0–40 cm soil layer, residual NO_3_^-^–N followed the order R > UR > U, whereas in the 0–120 cm layer, it followed the order U > R > UR. These findings align with previous research (Xiao et al., 2019), which reported that R can reduce N leaching but may also increase the NO_3_^-^–N content in the topsoil (0–40 cm). Under U, NO_3_^-^–N was primarily concentrated in the 60–120 cm deep layer, indicating substantial downward leaching. This distribution pattern is comparable to that commonly observed in local conventional farmland, where urea is typically applied at higher rates (Liu et al., 2023). A recent study in the Ningxia Yellow Irrigation District of Northwest China reported that the local farmers’ conventional N application rate reached 420 kg N ha^−1^, far exceeding the crop’s actual requirement (Wang Y. et al., 2025). In such high-N-input systems, NO_3_^-^–N is frequently leached below the root zone (60–120 cm) due to the rapid nitrification of urea-N and heavy rainfall events during the summer maize season (Li Z. et al., 2020). It is well established that reducing excessive N fertilizer application can mitigate the risk of N pollution in soil (Liu et al., 2021). In this study, R had significantly lower residual NO_3_^-^–N in the 0–120 cm soil layer than U, but still higher than UR. This may be due to the slower release rate of R at the early maize growth stages, which failed to meet maize N demand during this critical period (Zheng et al., 2020). As a result, maize growth and NUE were reduced, leading to increased NO_3_^-^–N accumulation in soil. Furthermore, residual NO_3_^-^–N in the 0–120 cm soil layer followed the order UR1 > UR3 > UR2. This suggests that UR2, with its more uniform N release, may have improved the temporal alignment between N supply and crop demand resulting in reduced residual NO_3_^-^–N in the soil compared with other ratios (El-Sharkawy et al., 2024; Shi et al., 2024). Moreover, the amount and distribution of rainfall during the growing season significantly influenced the soil NO_3_^-^–N content (Li Z. et al., 2020). Studies have shown that NO_3_^-^–N in the soil is more prone to leaching into deeper layers (> 120 cm) during heavy rainfall, which may explain the higher NO_3_^-^–N levels in deeper observed in this study (Yu et al., 2019). In this study, the cumulative N leaching and residual soil NO_3_^-^–N were significantly lower in UR2 than in UR1 and UR3. This may be because the moderate U/R ratio (3/7) in UR2 improved the temporal correspondence between N supply and crop demand throughout the growing season (Fu et al., 2026). Therefore, appropriately adjusting the blending ratio of U and R can help reduce soil N residue and mitigate the potential risk of N leaching. It should be noted that soil NO_3_^-^–N content was measured only at harvest in this study, which provides a useful endpoint assessment but does not capture the dynamic changes in nitrate concentration across different growth stages. Multi-stage sampling (e.g., at seedling, jointing, silking, and maturity stages) would provide stronger evidence for N release–uptake synchrony and nitrate movement through the soil profile (Akhtar et al., 2025). Future studies should combine soil NO_3_^-^–N monitoring at multiple growth stages to more precisely quantify N leaching losses under different blending ratios.

Previous studies have demonstrated that differences in the types and concentrations of root exudates among crop genotypes can influence rhizosphere microbial communities (Li et al., 2022). These microbial communities, in turn, affect NUE through their involvement in N fixation, transformation, and mineralization (Matus-Acuña et al., 2021). Moreover, differences in root architecture and spatial distribution between maize genotypes directly affect the crop’s N uptake capacity (Ibn et al., 2024). These factors may partly explain why the NO_3_^-^–N residue in QL14 was lower than in ZD958. Together with the lower NH_3_ volatilization observed in QL14, these results indicate that QL14 exhibits a more efficient N acquisition and utilization system. Other studies have also indicated that differences in root exudates among cultivars influence the growth of beneficial microorganisms, thereby affecting N transformation, uptake, and NO_3_^-^–N accumulation (Parasar et al., 2024). Furthermore, N-efficient maize cultivars can utilize soil N resources more efficiently, reducing N residues in the soil (Yadav et al., 2023). Compared with local conventional farming practices, the combination of the 3/7 blending ratio (UR2) and QL14 cultivar substantially reduced NO_3_^-^–N residue in the deeper soil layers. This finding is consistent with Cao et al. (2024), who reported that blending coated urea with conventional urea at a 3/7 ratio reduced NO_3_^-^–N residue in the 100–200 cm soil layer while maintaining or slightly increasing crop yield. This practical advantage highlights the potential of the UR2 + QL14 strategy as a locally adaptable solution for improving N management and reducing nitrate leaching risk in rainfed maize production systems.

### 4.3 Effects of fertilizer type and N application rate on maize growth, yield and N use efficiency

N fertilization plays a critical role in regulating maize yield formation and NUE (Jiang et al., 2024). In this study, the UR and R treatments resulted in higher yield and NUE than U. These findings are consistent with previous studies showing that blended N sources can improve the temporal match between N supply and crop N demand, thereby enhancing yield and NUE while minimizing N losses (Li G. et al., 2020; Liu et al., 2021). Specifically, U and blended treatments showed higher DMA, chlorophyll content, and *P*_n_ at early growth stages than R. This may be due to the rapid hydrolysis of U to NH_4_^+^–N, which is quickly available for maize uptake during early vegetative growth, thereby supporting initial growth and promoting biomass accumulation (Zheng et al., 2020). However, at later growth stages, the gradual N release characteristics of the R and UR treatment led to higher DMA, *P*_n_, and yield, suggesting that these treatments may have provided a more sustained N supply throughout the growing season, particularly during reproductive growth (Dai et al., 2022; Lawrencia et al., 2021). In this study, DMA, LAI, grain yield, and NUE were generally higher under N1 than under N2, which is consistent with previous findings (Srivastava et al., 2018). Excessive N application can lead to imbalances in nutrient uptake and reduced physiological efficiency, potentially diminishing NUE and growth performance (Zhang et al., 2022c).

The study also highlighted cultivar-specific differences in growth and NUE. QL14 exhibited better DMA performance than ZD958 across different N fertilizer types and application rates, which could be attributed to genetic differences in N uptake and utilization efficiency, as reported in previous studies (Ibn et al., 2024; Theeuwen et al., 2022). The higher N remobilization efficiency observed in QL14 aligns with findings from a recent study by Tian et al. (2026), who reported that a 70% controlled-release urea treatment (C70) significantly increased leaf and stem N accumulation at tasseling and maintained higher ear N during grain filling compared with conventional urea treatments, thereby enhancing post-silking N remobilization. In that study, the C70 treatment achieved a 10.07% increase in NUE compared with conventional split urea application and reduced nitrate concentrations in deeper soil layers. Our results are consistent with these findings, as UR2 similarly improved N uptake and remobilization parameters (VNR and NRE) compared with other blending ratios and conventional urea. To further elucidate these differences, Mantel test and Pearson correlation analysis were employed to examine the relationships among soil N residues, physiological traits, yield, and NUE. The results revealed that the correlations were generally stronger and more extensive for QL14 than for ZD958. Notably, in QL14, yield and NUE exhibited significantly stronger positive correlations with post-silking N uptake (PSN), and yield was also significantly positively correlated with LAI, whereas such relationships were weaker in ZD958. Furthermore, cumulative NH_3_ volatilization and NO_3_^-^–N residue showed more extensive negative correlations with key physiological traits (e.g., DMA, *P*_n_) and NUE components in QL14. These results indicate that QL14 possesses a more integrated physiological system linking N uptake, photosynthetic capacity, and canopy structure, enabling a stronger N-driven growth response and leading to lower N losses and higher NUE under the same N input (Yan et al., 2022). Overall, the correlation network among indicators in QL14 was denser and stronger than that in ZD958, particularly for yield and NUE, which showed strong correlations with NH_3_ and NO_3_^-^–N.

To identify the optimal fertilizer management strategy, an entropy-weight TOPSIS comprehensive evaluation model was applied, integrating 14 indicators across all treatments. The results showed that the ranking trends of the comprehensive scores were highly consistent between the two varieties, with UR2N1 (3/ 7, 180 kg·ha^−1^) ranking as the top treatment for both cultivars, achieving comprehensive scores of 0.811 for ZD958 and 0.817 for QL14. Therefore, UR2N1 is identified as the optimal N fertilizer management strategy under the conditions of this experiment, and QL14 demonstrates a more favorable physiological and agronomic response to this strategy compared to ZD958. Although slow-release fertilizers are usually more expensive than common urea, the grain yield under the UR2N1 strategy is 46–56% higher than that of urea alone. In addition, the one-time basal application of the UR mixture reduces the labor and time costs associated with multiple topdressings required for conventional urea (Zhang et al., 2022b). Previous studies have also shown that, compared with traditional urea, the optimized blending ratio can increase the net profit by 8.50–15.20%, indicating that the yield gain from UR2N1 may offset the higher material cost (Shi et al., 2024; Sun et al., 2024).

It is important to note that this study was conducted during a single growing season (2018) at one experimental site in Northwest China. The 2018 growing season received 482 mm of precipitation and had a mean temperature of 22.4 °C during the maize growing period, which were close to the 10-year averages (456 mm and 22.1 °C) for this region, supporting the representativeness of our findings for typical years. However, the release kinetics of polymer-coated slow-release fertilizers are highly sensitive to soil temperature and moisture (Fan et al., 2011; Lawrencia et al., 2021). Therefore, years with above average rainfall or extreme temperature events may accelerate or delay the release of N in slow-release fertilizers, which may alter the optimal blending ratio determined in this study. Although our results provide reliable evidence under the specific conditions of the site in 2018, multi-year and multi-site experiments are needed to verify the UR2N1 strategy and evaluate its stability under different agro-ecological conditions. In addition, only two maize varieties (ZD958 and QL14) were investigated in this study; whether the observed variety-specific response to the UR mixture ratio extends to other widely cultivated genotypes warrants further investigation. Finally, our assessment of N loss is mainly focused on the NH_3_ volatilization; future research should also quantify N_2_O emissions and nitrate leaching, and measure fertilizer N release kinetics to more comprehensively evaluate the environmental benefits of UR mixtures and confirm the inferred synchronization mechanism.

## 5 Conclusions

Compared with urea (U) and slow-release N fertilizer (R), blended application (UR) generally helped improve maize grain yield and NUE while reducing environmental N losses. Among all tested treatments, the UR2N1 (3/7, N1: 180 kg·ha^−1^) performed the best. Under the UR2N1, grain yields of ZD958 and QL14 reached 7610.57 and 8573.14 kg·ha^−1^, respectively, and NUE increased by 3.51–19.07% and 4.01–20.41% compared with other treatments. In addition, NH_3_ volatilization and soil NO_3_^-^–N residue were also significantly reduced under UR2N1. Meanwhile, UR2N1 significantly promoted N uptake and remobilization in maize, with N remobilization efficiency (NRE) increased by 3.37–109.13%. Compared with ZD958, the N-efficient cultivar QL14 exhibited higher grain yield (12.65%) and NUE (9.70%), as well as lower soil NO_3_^-^–N residue (8.78%) and NH_3_ volatilization (14.12%) under the same fertilization. These findings indicate that the UR2N1 and QL14 combination can enhance maize yield and NUE while reducing environmental N losses, effectively balancing the trade-off between grain yield and environmental impact, and can be recommended as an appropriate fertilization–cultivar strategy for rainfed maize production in Northwest China. It should be noted that these findings are based on a single growing season (2018) at one experimental site. Although the climatic conditions of that year were representative of normal years for this region, the stability of the optimal strategy and its generalizability to other environments require further validation through multi-year and multi-location trials.

## Data Availability

The original contributions presented in the study are included in the article/**Supplementary Material**, further inquiries can be directed to the corresponding author/s.
