# Supplementary material for: Optimizing the blending ratio of urea with slow-release N fertilizer improves maize yield and N use efficiency while reducing N losses
Source: Front Plant Sci. 2026 Jul 28;17:1895413. doi: 10.3389/fpls.2026.1895413 (PMC13457041; doi:10.3389/fpls.2026.1895413)
Supplement: Supplementary file 1 [file Supplementaryfile1.docx]

Supplementary Material

# 1 Supplementary Figures

**Figure S1.** Annual mean temperature, relative humidity, precipitation, and sunshine duration at the experimental site (2009–2018).
